# Successful Management of Peri-Implant Infection from the Endodontic Lesion of Adjacent Natural Tooth

**DOI:** 10.1155/2023/5034582

**Published:** 2023-03-14

**Authors:** Jiaming Gong, Abeer A. Al-Sosowa, Ruimin Zhao, Jianxue Li, Mei Mei

**Affiliations:** ^1^Department of Stomatology, The 940th Hospital of Joint Logistics Support Force of People's Liberation Army, Lanzhou, Gansu, China; ^2^Department of Stomatology, Quzhou Hospital Affiliated to Wenzhou Medical University (Quzhou people's Hospital), Quzhou, Zhejiang, China; ^3^Department of Periodontics, Faculty of Dentistry, Thamar University, Dhamar, Yemen

## Abstract

Recently, dental implants have had the most important role in oral rehabilitation. Peri-implantitis is considered a common complication of dental implants. Adjacent natural teeth with untreated endodontic pathology may be a potential risk for implant placement. Retrograde/periapical peri-implantitis (RPI), the inverting of the progress direction of peri-implantitis. Radiographically, it is characterized by signs of periapical bone loss and normal coronal osteointegration of the implant; and its prevalence is closely associated with endodontic lesions of adjacent teeth. Another novel separate disease entity is known as the endodontic peri-implant defects (endo-implant defects), manifesting as the peri-implant marginal bone loss due to endodontic pathology of adjacent teeth, to which endodontists and implantologists are supposed to attach great importance. This current study presented two cases of different types of peri-implant infection in which conducting proper intervention to the endodontic lesions of adjacent teeth resulted in full radiographic and clinical resolution of peri-implant defects.

## 1. Introduction

Intraosseous implants, possessing the advantage of not affecting the integrity of adjacent teeth and the aesthetic properties, have frequently been adopted to restore dentition defects since the concept of osseointegration was proposed [1]. However, even strictly observing the surgical protocols, their widespread clinical practice led to implant failure for multiple causes including: iatrogenic factors (bone overheating or osteonecrosis during osteotomy, inaccurate location, and surgical trauma), excessive load, and peri-implantitis. Another contributing factor of peri-implant infection is associated with endodontic lesions of the adjacent teeth, e.g., retrograde peri-implantitis (RPI) and endodontic peri-implant defects (endo-implant defects).

RPI was first described in 1992 by McAllister et al. [2] and formally defined by Reiser et al. [3] in 1995 as an infectious lesion developing in the implant apex during osseointegration and maintaining normal coronal bone in the early stage, which can be diagnosed by radiography [4]. It is believed to be associated with endodontic lesions of adjacent teeth. And its prevalence increased from 0.26% to 25% when endodontic lesions of adjacent teeth were present [4–6]. RPI is often accompanied by clinical signs of inflammation, including swelling, pain, or sinus tract around the implant apex, but it may also present without any sensation.

Endo-implant defects were first proposed by Daubert et al. as a coronal peri-implant pathology caused by an endodontic lesion of the adjacent tooth [7]. It usually shows similar manifestations to peri-implantitis, such as mucositis and marginal bone loss. However, radiography can distinguish between peri-implantitis and endo-implant defects, which have an infectious penetrating between adjacent tooth and implant.

The above two separate disease entities expand the current categories of peri-implant diseases. Furthermore, describe the dynamic interaction between the implant and endodontic microenvironment, which needs to raise particular concerns among endodontists and implantologists. Clinically, recognition of early clinical signs and symptoms, regular radiographic evaluation, and reasonable intervention can minimize the extent of peri-implant destruction.

This report describes two cases of different types of peri-implant diseases originating from periapical periodontitis in adjacent teeth. In case 1, RPI was successfully resolved by extraction of the affected tooth, and a biopsy of the affected tissue around the implant was consistent with an inflammatory granuloma. In case 2, the source of infection in the adjacent tooth was traced by the combination of a gutta-percha cone and periapical radiograph, and the endo-implant defect was cured by root canal treatment (RCT) of the adjacent tooth. The bacterial culture of pyogenic fluids confirmed that the dominant bacteria were Streptococcus.

## 2. Case Presentation

### 2.1. Case One

**Figure 1:**
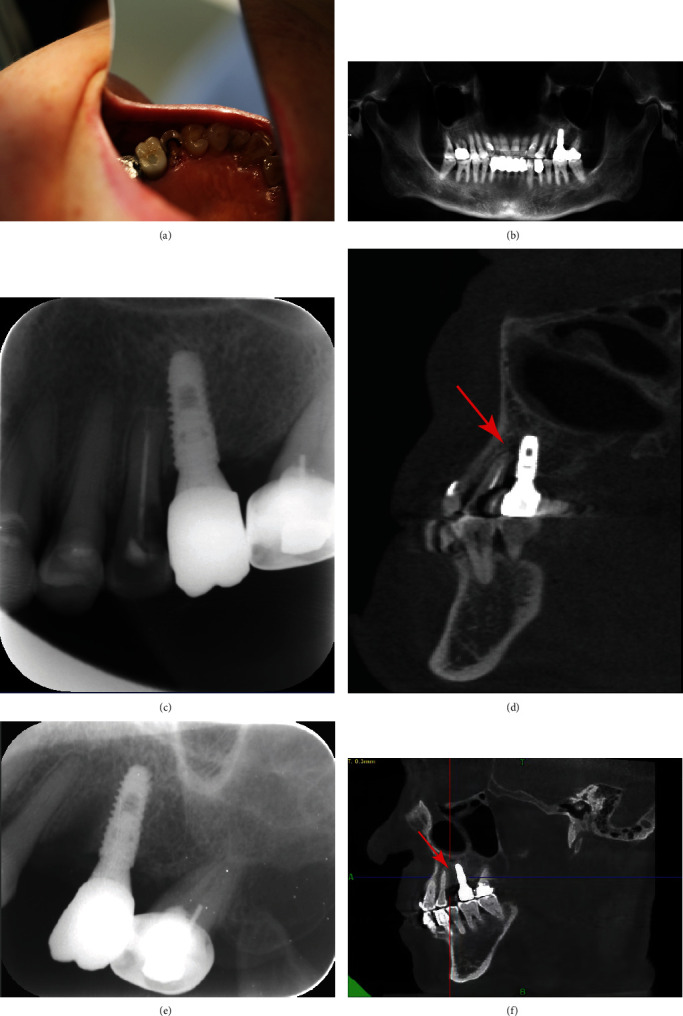
Case 1: (a) Preliminary photograph showed a defect of the palatal crown to the subgingival at tooth #25. (b) The original Cone-beam CT. (c) The apical radiograph revealed the insufficient distance between tooth #25 and the implant. (d) The coronal radiograph clearly showed that the apical lesion of tooth #25 destroyed the peri-implant bone (red arrow). (e) Three months after extraction. (f) Intraoral radiography one year after extraction (red arrow).

**Figure 2:**
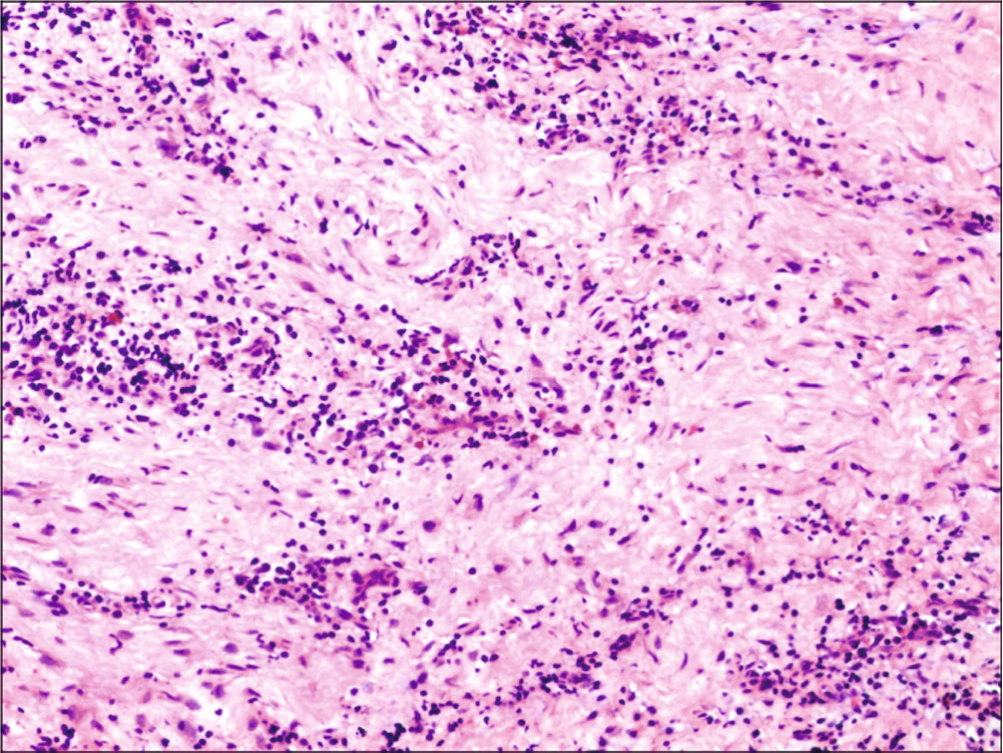
Histological evaluation of peri-implant tissue revealed infiltration of lymphocytes and plasma cells (×100).

A 72-year-old woman presented to the Joint Logistic Support Force Hospital with split teeth while eating. She claimed to have a history of controlled hypertension and no smoking habit. Intraoral examination revealed that the palatal crown of tooth #25 fractured to the subgingival, resulting in exposure of the endodontic cavity (Figure 1(a)). Apical radiography of tooth #25 indicated that the distance between implant #26 and tooth #25 was insufficient, and inappropriate RCT of tooth #25 (Figure 1(c)). Clinical probing of tooth #5 ranged from 3 to 5 mm and implant #6 from 2 to 3 mm. Cone-beam CT was used for a more comprehensive evaluation (Figure 1(b)); the apical infection in tooth #25 had spread to the implant tip; however, the patient claimed no clinical symptoms, even when tooth #25 and implant #26 were percussive. Because of the adverse crown defect and the uncertainty of retreating tooth #25, it was extracted directly under local infiltration anesthesia. Both the inflammatory tissue in the extraction socket and tissue penetrating the implant tip were curette (plastic implant scalers). Moreover, a biopsy was performed; a large amount of plasma cell and lymphocyte infiltration was observed under the microscope, consistent with inflammatory granuloma (Figure 2). The implant apex and extraction socket were repeatedly rinsed with gentamicin solution (2 ml, Tiandi Pharmaceutical Co., Ltd., Guizhou, China) and saline through a 5 ml syringe to eliminate as much potential infection as possible. Radiographic follow-up of the patient at 3 and 12 months revealed resolution of the defect through increasing the density around the implant and within the extraction socket (Figures 1(e) and 1(f).

### 2.2. Case Two

**Figure 3:**
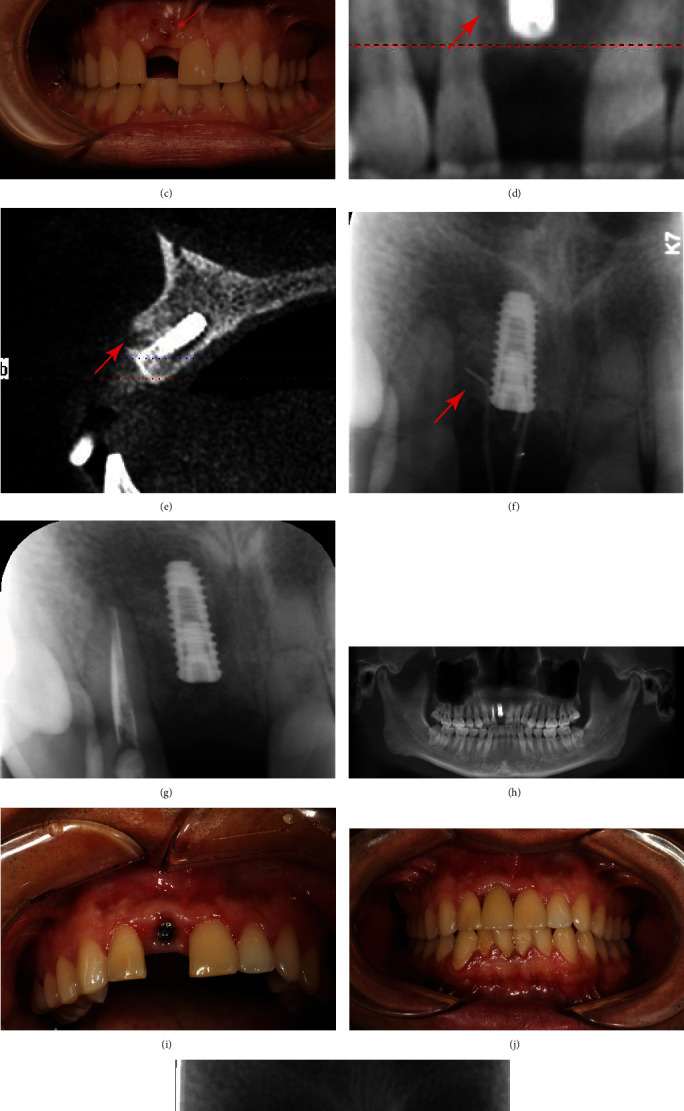
Case 2: (a) Preliminary Cone-beam CT before implant placement. (b) A coronal image of site #11 indicated the absence of labial bone and poor socket healing. (c) Fistulas were observed in the labial mucosa around implant #11(red arrow). (d, e) Follow-up visit after implant placement for 5 months, radiography appeared to indicate a mesial spread of apical periodontitis in tooth #12 to the coronal peri-implant defect (red arrow). (f) Apical radiograph revealed a diagnostic Gutta-percha cone tracing from mucosal fistulas to tooth #12 (red arrow). (g) Periapical radiographs at 1-month follow-up after filling the root canal with Vitapex. (h) Cone-beam CT indicated that RCT of tooth #12 was suitable, and the peri-implant bone defect was healed after half a year. (i) Intraoral photographs half a year after root canal filling. (j) The intraoral photograph of the implant-supported restoration 7 months after RCT showed healthy peri-implant mucosa. (k) Apical radiograph showed no defects of peri-implant supporting bone. Root canal therapy, RCT.

A 24-year-old male serviceman was referred to an implantologist for the implant therapy of a traumatic maxillary incisor extraction (Figures 3(a) and 3(b)). The patient was healthy without any dental disease record. One bone-level endosseous 3.5 × 11.5 mm implant (Osstem, Seoul, Korea) was accurately embedded in the extraction socket. 0.25 g bovine bone particles (Bio-Oss®; Geistlich, Wolhusen, Switzerland) and absorbable collagen barrier membrane (Bio-Gide®; Geistlich, Wolhusen, Switzerland) were placed on the peri-implant defect site, and tension-free primary closure was achieved. Postoperatively, the patient was prescribed antibiotics (metronidazole 250 mg and amoxicillin 500 mg), three times daily for 3 days and ibuprofen (800 mg as needed for pain).

**Figure 4:**
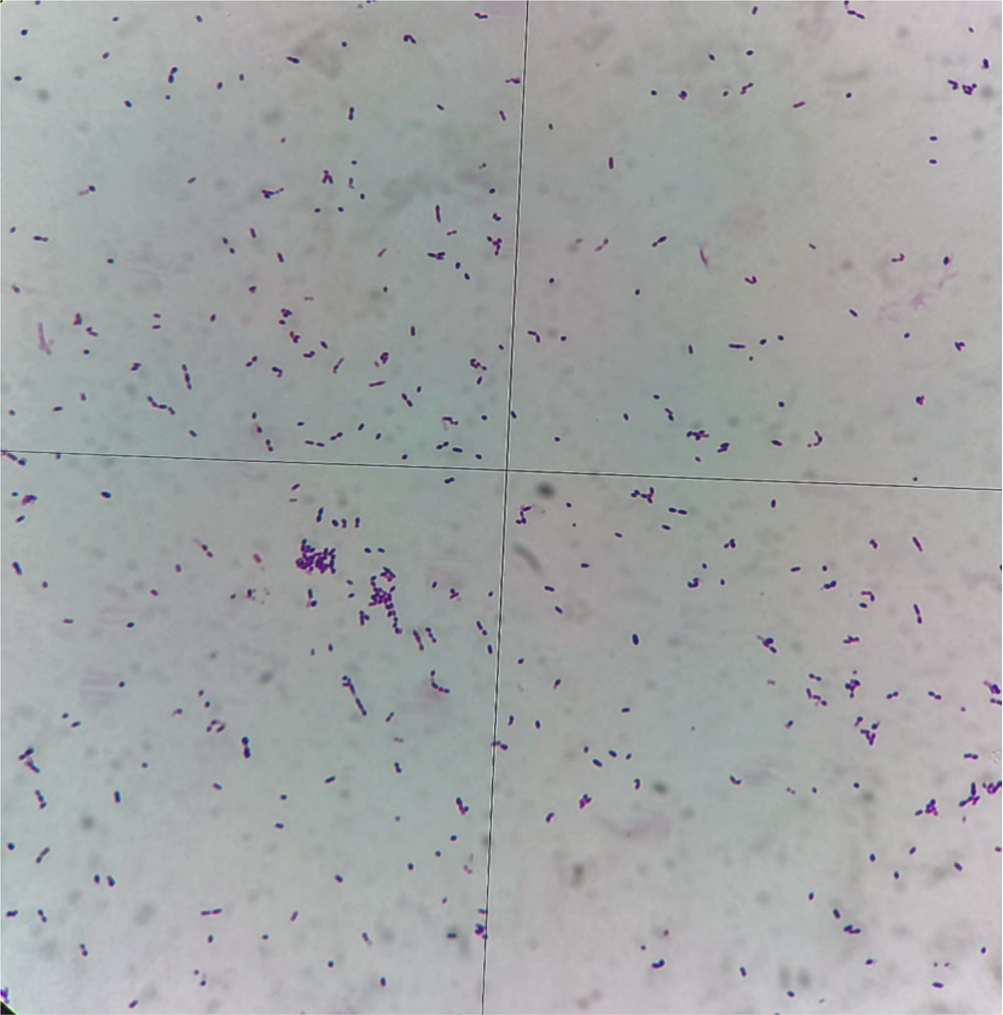
The bacterial culture of peri-implant tissues showed Streptococcus as the main pathogenic bacteria. (Gram staining, ×100).

Five months later, fistulas appeared in the facial mucosa at site #11 while preparing for the second stage procedure (Figure 3(c)). A probing examination showed approximately 5 mm of bone loss localized to the interproximal region between implant #11 and tooth #12. Radiography indicated a mesial spread of apical periodontitis in tooth #12 to the coronal peri-implant defect (Figures 3(d) and 3(e)). Diagnostic gutta-percha cone running along the fistulas indicated tooth #12 as the cause (Figure 3(f)). Part of the purulent exudate through the labial fistula was collected and cultured for 72 hours. Streptococcus was observed under the microscope (Figure 4). Therefore, control of periapical infection in tooth #12 is considered to be the preferred option, and surgical management was decided if the result was not satisfactory.

Access to tooth #12 was created, and one canal was located. Shaping and cleaning the canal to the working length via a standard crown-down technique were performed using rotary and hand ProTaper files (Dentsply, United States). Irrigation was performed with 5.25% sodium hypochlorite and 17% Ethylene Diamine Tetraacetic Acid (EDTA). Followed by paper points drying and placement of Vitapex (Morita, Japan) as intracanal medicament. Amoxicillin was given to prevent infection after the operation (500 mg three times daily for 7 days).

The mucosal healing around the implant and the periapical lesion of tooth #12 healed at the second visit, 4 weeks after the initial visit of RCT (Figure 3(g)). The canal was reinstrumented and reirrigated, as previously mentioned. Then subsequently dried and obturated with canal sealer (AH PLUS; Dentsply, United States) via a single gutta-percha cone technique. The tooth was temporized with Cavit (3M ESPE, United States). Meantime, the healing cap was successfully installed after raising the full-thickness flap to confirm that the supporting bone was healthy. Follow-up after half a year with radiographs found healing evidence of the periapical radiolucency around the tooth apex #12 and the coronal part of the implant (Figures 3(h) and 3(i)). One month later, the implant was permanently restored with a cemented crown (Figures 3(j) and 3(k)).

## 3. Discussion

With the popularization of dental implants and standardized management of endodontic diseases, the relationship between implants and adjacent teeth has to be a major concern. The interaction between adjacent teeth and the implant is mostly revealed in RPI, which is divided into implant-to-tooth and tooth-to-implant according to the pathogenesis by Sussman [8]. Implant-to-tooth is when the implant insertion results in an endodontic pathology of the neighboring tooth (e.g., insufficient distance, bone overheating/osteonecrosis during osteotomy), which may influence the osseointegrated implant. Tooth-to-implant is interpreted as the presence of adjacent teeth with risk factors for endodontic lesions such as root resorption, caries, or trauma. Bacteria from adjacent teeth spread through the interosseous space and colonize closer to the implant [9]. Case 1 accords with tooth-to-implant, where inappropriate RCT of the neighboring tooth caused apical periodontitis, and insufficient distance provided a favorable environment for infection expansion.

There is currently no valid classification of endo-implant defects, but case 2 may be more suitable for tooth-to-implant regarding the above classification. The devitalization of adjacent teeth may have occurred before implantation because of trauma. After implant placement, the damage/cutting off of the blood supply of the pulp cavity may further aggravate the inflammation, resulting in periapical infection [8].

RPI resembles endo-implant defects, in that, both are site-specific infective processes, with the main differences lying in the pathway of infection and microbial composition. RPI initiates apically, whereas endo-implant defects occur coronally. Therefore, the former relies on the patient complaint and radiographic evaluation, whereas the latter can be detected clinically via routine probing. Furthermore, microorganisms found in RPI are much related to the source of infection (e.g., residual infection at the implant site due to previous apical periodontitis, refractory apical periodontitis, cyst formation), mainly for *Porphyromonas gingivalis*, Corynebacterium, Streptococcus, and *Klebsiella pneumoniae* [10]. But microbes of endo-implant defects are relatively uncomplicated, only associated with the endodontic complication of adjacent teeth. In case 2, purulent culture from peri-implant fistula found Streptococcus, the causative bacteria of periapical periodontitis [11], which supported our suspicion. In the control of infection, metronidazole can effectively resist the toxins produced by Streptococcus to some extent [12]. However, no suppuration was collected in case 1, and bacterial culture was challenging to complete. Therefore, the diseased tissue around the implant was collected for biopsy when the affected tooth was extracted, and the discovery of inflammatory granuloma also reflected that bacteria contaminated the implant [13].

Infection from adjacent teeth may lead to implant failure if incorrect diagnosis and untimely intervention. Conventional radiological methods, including apical radiography and cone-beam CT, are recommended, focusing on implants and adjacent anatomical tissues. In case 2, diagnostic gutta-percha was used to further investigate the source of infection, and peri-implantitis was finally excluded, which is of instructive value in endo-implant defects with fistula [14].

It is critical to effectively remove the source of infection after identifying the cause, and progressive management instead of immediate invasive surgery seems to be prudent and appropriate. Both cases followed this principle, and individualized treatment was taken according to the status of adjacent teeth, including tooth extraction, RCT, and apicoectomy [5, 15]. Radiologically, the density of the defect around the implant increased without sign of infection recurrence. It is reasonable to think that the infection control of adjacent teeth has received good feedback, and there is no surgical indication for surgical debridement. Some scholars [7] have proposed preventive debridement while managing endodontic complications, including potentially infected tissue, implant surface detoxification, and reconstruction of the lost host tissue. This method seems to reduce the recurrence of peri-implant infection, but it is not friendly to implant surface damage, material expenditure, and patient feelings. To the best of our knowledge, mechanical preparation combined with antimicrobials is effective in removing periapical infection of adjacent teeth (gradually shaping the inner wall of canals, rinsing with a high concentration of sodium hypochlorite and EDTA) [16]. It is suggested that more active intervention measures should be considered when RCT is ineffective [15].

Peri-implant infection originating from endodontic disease is a preventable disease. Proper radiographic assessment for potential or existing pathology of implant sites and adjacent tissues and prophylactic use of antibiotics after implantation must be considered.

## 4. Conclusion

As the etiology of peri-implant infection is related to the adjacent endodontic disease, it is beneficial for the implant's prognosis that endodontists can collaborate with implantologists. Meanwhile, surgical therapy is considered to be prudent, and prioritizing the non-surgical protocol for adjacent endodontic lesions may be more reasonable. More critically, the teeth adjacent to the planned implant site should be advocated for careful evaluation and advanced intervention to potential endodontic disease by endodontists.

## Data Availability

The study data will be available upon request to the corresponding author.
